# Potential Role of Mic60/Mitofilin in Parkinson’s Disease

**DOI:** 10.3389/fnins.2018.00898

**Published:** 2019-01-25

**Authors:** Victor S. Van Laar, P. Anthony Otero, Teresa G. Hastings, Sarah B. Berman

**Affiliations:** ^1^Department of Neurology, School of Medicine, University of Pittsburgh, Pittsburgh, PA, United States; ^2^Pittsburgh Institute for Neurodegenerative Diseases, University of Pittsburgh, Pittsburgh, PA, United States; ^3^Division of Neuropathology, Department of Pathology, School of Medicine, University of Pittsburgh, Pittsburgh, PA, United States; ^4^Cellular and Molecular Pathology (CMP) Program, Department of Pathology, School of Medicine, University of Pittsburgh, Pittsburgh, PA, United States; ^5^Department of Neuroscience, University of Pittsburgh, Pittsburgh, PA, United States; ^6^Clinical and Translational Science Institute, University of Pittsburgh, Pittsburgh, PA, United States

**Keywords:** Mic60/mitofilin, mitochondria, Parkinson’s disease, neurodegeneration, mitochondrial dynamics

## Abstract

There are currently no treatments that hinder or halt the inexorable progression of Parkinson’s disease (PD). While the etiology of PD remains elusive, evidence suggests that early dysfunction of mitochondrial respiration and homeostasis play a major role in PD pathogenesis. The mitochondrial structural protein Mic60, also known as mitofilin, is critical for maintaining mitochondrial architecture and function. Loss of Mic60 is associated with detrimental effects on mitochondrial homeostasis. Growing evidence now implicates Mic60 in the pathogenesis of PD. In this review, we discuss the data supporting a role of Mic60 and mitochondrial dysfunction in PD. We will also consider the potential of Mic60 as a therapeutic target for treating neurological disorders.

## Introduction

Parkinson’s disease (PD), the most common neurodegenerative movement disorder, was first described in 1817 by James Parkinson in “An Essay on the Shaking Palsy.” (Parkinson, 1817) In the 200 years that have passed since recognition of this neurological disorder, great strides have been made to characterize disease pathology, distinguish clinical symptoms, and develop a therapeutic treatment. However, the causes of PD neurodegeneration are still unknown, *and there is no cure nor are there any available neuroprotective therapies to hinder disease progression*. Identifying and understanding the etiology of PD progression is key to the development of new therapeutics for disease treatment.

Epidemiological studies and laboratory research have long sought to find potential causes of this prevalent disease (Khandhar and Marks, 2007; Delamarre and Meissner, 2017). Though the underlying mechanism of PD pathogenesis remains elusive, current ideology suggests that a combination of environmental exposure and genetic predisposition are responsible for most cases of PD (Horowitz and Greenamyre, 2010; Ritz et al., 2016). Genetic and epidemiological studies have identified multiple biological pathways that promote PD pathogenesis, many of which converge on the function of the mitochondria, the “powerhouses” of the cell (Schapira, 2008; Cieri et al., 2017; Ammal Kaidery and Thomas, 2018; Zanon et al., 2018).

**FIGURE 1:**
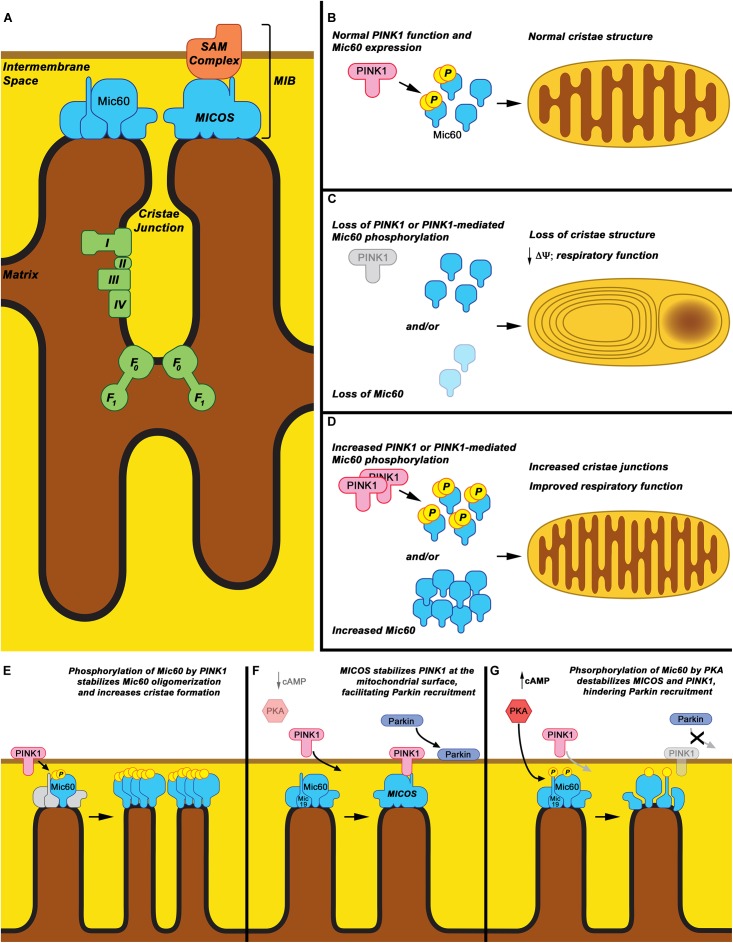
Mic60: Mitochondrial cristae structure maintenance and proposed phospho-regulation. **(A)** Mic60 is an inner mitochondrial membrane protein and key component of the MICOS and MIB protein complexes, which bridge inner membrane cristae junctions and inner-outer membrane contact sites, respectively. Maintenance of cristae structure by MICOS also regulates function of the mitochondrial Electron Transport Chain Complexes (I, II, III, and IV) and ATP synthase (F_0_-F_1_). **(B)** It is proposed that under normal conditions, Mic60 interacts with and is phosphorylated by PINK1 at a basal rate, thus regulating its ability to maintain normal cristae structure. **(C)** Under conditions in which PINK1 expression is decreased, PINK1 kinase function is inactivated, or Mic60 expression is reduced, the mitochondrial cristae structure is not maintained. This leads to mitochondria exhibiting characteristic onion ring-like whorls of the inner membrane or formation of large vacuoles within the mitochondrion. This is accompanied by decreased membrane potential (Δψ) and respiratory function. **(D)** Under conditions in which PINK1 phosphorylation of Mic60 is increased or Mic60 expression is increased, mitochondria can exhibit increased numbers of inner membrane cristae structures and cristae junctions. This is accompanied by highly coupled respiratory function. **(E)** One proposed mechanism of PINK1’s effects on Mic60 is that phosphorylation increases the ability of Mic60 to oligomerize with itself and, presumably, the MICOS complex, leading to increased inner membrane structural integrity. **(F)** In a cellular state in which cAMP levels and PKA activation are low, PINK1 can also interact with Mic60 and be stabilized on the surface of mitochondria, aiding in its function to recruit Parkin to damaged mitochondria. **(G)** However, in apparent opposition to the PINK1-Mic60 interaction, PKA activation and phosphorylation of Mic60 destabilizes the MICOS complex and decreases the ability of PINK1 to stabilize on the mitochondrial surface, preventing the recruitment of Parkin to damaged mitochondria.

Mitochondrial dysfunction is a known contributor to PD pathophysiology, with impaired mitochondrial respiration, morphology, and fission/fusion/transport dynamics all associated with PD (Van Laar and Berman, 2013; Bose and Beal, 2016). The connection between PD and mitochondria is reinforced by heritable forms of the disease, wherein monogenetic PD-causing mutations in nuclear-expressed proteins such as PINK1, Parkin, LRRK2, and alpha-synuclein have all been shown to affect mitochondrial function (Narendra et al., 2010; Sanders et al., 2014; Di Maio et al., 2016; Verma et al., 2017). In recent years, independent studies from multiple labs have associated the inner mitochondrial membrane protein Mic60, also known as mitofilin, with PD pathogenesis (Van Laar et al., 2008, 2009, 2016; Akabane et al., 2016; Tsai et al., 2018). Mic60 is a core component of the mitochondrial contact site and cristae junction organizing system (MICOS) (Zerbes et al., 2012b; Pfanner et al., 2014; Kozjak-Pavlovic, 2017). The MICOS is a large, multi-protein complex of the mitochondrial inner membrane that maintains cristae structure, forms inner-outer mitochondrial membrane contact sites, organizes respiratory complexes, and regulates protein import (Bohnert et al., 2012; Zerbes et al., 2012a; Harner et al., 2014; Pfanner et al., 2014; Friedman et al., 2015; Horvath et al., 2015; Kozjak-Pavlovic, 2017; Figure 1A). Growing evidence places the MICOS complex, and in particular the protein Mic60, in a central role in regulating PD-relevant mitochondrial processes. Here, we will review the role of Mic60 in mitochondrial function and will review evidence for a role for Mic60 in PD neurodegeneration and as a potential therapeutic target in PD.

## Mitochondrial Dysfunction, Mitochondrial Dynamics, and Oxidative Stress in PD

PD is a progressive neurodegenerative disorder. A pathological hallmark of PD is a loss of the dopamine (DA) neurons of the nigrostriatal pathway, though other populations throughout the midbrain, basal ganglia, and cortex degenerate as well (Braak et al., 2003, 2004). Despite years of study, the cause of this neurodegeneration is still unknown, but growing evidence implicates mitochondrial respiratory dysfunction, oxidative stress, and dysregulation of mitochondrial dynamics in the neuropathogenesis of PD, as has been thoroughly reviewed elsewhere (Murphy et al., 1999; Toescu et al., 2000; Friberg and Wieloch, 2002; Beal, 2007; Schapira, 2008; Van Laar and Berman, 2009; Exner et al., 2012; Cieri et al., 2017; Ammal Kaidery and Thomas, 2018). To briefly summarize, many studies have identified protein oxidation, DNA damage, phospholipid oxidation, and decreased function of mitochondrial respiration in brain tissue from PD patients (Dexter et al., 1989; Schapira et al., 1990; Alam et al., 1997a,b; Good et al., 1998; Sanders and Greenamyre, 2013). A low-grade deficiency in mitochondrial electron transport chain (ETC) Complex I (NADH dehydrogenase) activity has been found not only in PD brain, but also other non-neuronal tissues throughout the body (Parker et al., 1989, 2008; Schapira et al., 1989; Krige et al., 1992; Yoshino et al., 1992; Barroso et al., 1993; Mann et al., 1994; Taylor et al., 1994; Haas et al., 1995; Penn et al., 1995; Blandini et al., 1998; Keeney et al., 2006), suggesting a systemic mitochondrial respiration deficit associated with PD. The discovery of the DA neuron-specific toxicant N-methyl-4-phenyl-1, 2, 3, 6-tetrahydropyridine (MPTP) in the 1980’s further implicated mitochondria in PD (Davis et al., 1979; Langston and Ballard, 1983; Langston et al., 1983). MPTP toxicity is elicited through its metabolite MPP+, which is selectively imported into DA neurons and acts as a Complex I inhibitor (Nicklas et al., 1985; Ramsay et al., 1986; Mizuno et al., 1987). MPP+ results in increased production of ROS (Rossetti et al., 1988; Adams et al., 1993; Smith and Bennett, 1997) and a loss of nigrostriatal DA neurons (Langston et al., 1999), suggesting that environmental toxins affecting mitochondrial function could also contribute to PD. These discoveries ultimately led to the seminal finding that systemic administration of low doses of rotenone, also a Complex I inhibitor, leads to PD-specific neurodegeneration and pathology, despite rotenone freely crossing all cell membranes (Betarbet et al., 2000; Sherer et al., 2003; Cannon et al., 2009).

Mitochondrial Complex I is known to be the major site for production of reactive oxygen species (ROS). Complex I dysfunction or inhibition results in increased ROS production (Lenaz, 2001; Votyakova and Reynolds, 2001), implicating mitochondrially produced oxidative stress in PD pathogenesis. A role for ROS in pathogenesis is bolstered by the fact that many of the most susceptible neurons in PD contain dopamine (DA), which produces ROS through its metabolism and through oxidation to reactive quinones (Bindoli et al., 1992; Monks et al., 1992; Hastings et al., 1996; Hastings and Berman, 2000). DA quinones bind to sulfhydryl groups on free cysteine, glutathione, and protein cysteinyl residues in the cell, and DA oxidation products have been shown to alter mitochondrial respiration and permeability transition pore opening (Hastings et al., 1996; Berman and Hastings, 1999; Gluck et al., 2002; Gluck and Zeevalk, 2004).

In addition to mitochondrial respiratory deficiencies and oxidative stress, PD pathogenesis is associated with defects in the dynamic properties of mitochondria that maintain mitochondrial homeostasis (mitochondrial fission, fusion, transport, biogenesis, and degradation) and are necessary for maintaining bioenergetic function (for review, see (Chen and Chan, 2009; Van Laar and Berman, 2009; McCoy and Cookson, 2012; Van Laar and Berman, 2013; Bose and Beal, 2016). This evidence arises not only from studies of *in vitro* toxicant models of PD, but also from PD patient-derived cell lines, and actions of familial PD-causing gene products (Exner et al., 2007; Poole et al., 2008; Arnold et al., 2011; Bose and Beal, 2016). Perhaps the most well studied is the shared pathway of two PD-associated proteins PINK1 and Parkin, in which evidence suggests that Parkin works downstream of PINK1 to signal damaged mitochondria for autophagic degradation (Narendra et al., 2010; Pickrell et al., 2015).

The evidence suggests that regulation of mitochondrial respiratory, morphologic, and maintenance functions plays a critical role in PD pathogenesis. Proteins that integrate these various and interrelated mitochondrial structural and homeostatic functions are therefore uniquely positioned to play an important role in PD-relevant mitochondrial dysfunction. As we will detail below, Mic60 is emerging as central to these integrated mitochondrial functions and, importantly, in PD pathogenesis. Mic60 is integral in the maintenance of both structural dynamics and respiratory function of mitochondria and interacts with PD gene products. These functions place Mic60 in a unique position to regulate mitochondrial response to stress, particularly in mitochondria-dependent neurons, and increasing evidence, as detailed below, links Mic60 to PD pathogenesis.

## Mic60, a Protein at the Intersection of Regulation of Mitochondrial Function and Structure

Mic60 was first identified as “HMP,” heart muscle protein, due to its abundance in cardiac tissue (Icho et al., 1994). Later renamed “mitofilin” based on its structure and localization, subsequent studies demonstrated that human Mic60 is a nuclear-expressed mitochondrial protein that is targeted selectively to the inner mitochondrial membrane (Odgren et al., 1996; Gieffers et al., 1997). Human Mic60, which exists in both 88 kDa and 90 kDa isoforms, contains a cleavable mitochondrial targeting sequence, a transmembrane domain near the N-terminus that spans the inner mitochondrial membrane with the bulk of the protein jutting into the intermembrane space (Gieffers et al., 1997), and three coiled-coil domains characteristic of involvement in protein-protein interactions (Odgren et al., 1996; John et al., 2005).

John et al. (2005) first described Mic60/mitofilin as a critical protein for maintaining mitochondrial cristae structure and mitochondrial respiration. Perhaps the most remarkable characteristic that was noted in association with Mic60 was that loss of the protein resulted in the reorganization of the mitochondrial cristae structure. Mitochondria in Mic60/mitofilin-deficient cells exhibited concentric ring-like structures or whorls in place of the normal inner membrane cristae structure (John et al., 2005), an effect since noted by others in various cell and animal models with aberrant Mic60 expression (Rabl et al., 2009; Mun et al., 2010; von der Malsburg et al., 2011; Tsai et al., 2017; Tsai et al., 2018). John et al. also found that Mic60/mitofilin not only formed a homo-oligomeric structure with itself but also was present in a large multimeric protein complex (John et al., 2005). Shortly thereafter, Xie et al. demonstrated that Mic60/mitofilin associated with a protein complex including Sam50, coiled-coil-helix coiled-coil-helix domain-containing (CHCHD) proteins 3 and 6, and metaxins 1 and 2, proteins known to be involved in mitochondrial protein import and assembly (Xie et al., 2007), thus linking Mic60 to both structural and protein maintenance of the mitochondrion.

Subsequent studies confirmed that Mic60/mitofilin is indeed a core component of a larger functional multi-protein complex of the inner membrane, now known as the MICOS complex (Pfanner et al., 2014; Kozjak-Pavlovic, 2017). As previously noted, the MICOS complex is responsible for structural organization of the mitochondria. MICOS subcomplexes interact with mitochondrial membrane lipids to form cristae junctions and organize respiratory complexes; and interact with outer-membrane transport machinery to regulate mitochondrial protein import and biogenesis (von der Malsburg et al., 2011; Bohnert et al., 2012; Zerbes et al., 2012a; Harner et al., 2014; Pfanner et al., 2014; Ding et al., 2015; Friedman et al., 2015; Horvath et al., 2015; Eydt et al., 2017; Hessenberger et al., 2017; Rampelt et al., 2017; Tarasenko et al., 2017). A uniform nomenclature was established for the MICOS complex and its subunits Mic10 through Mic60, the name given to mitofilin (Pfanner et al., 2014). In metazoa, the MICOS complex also interacts with the sorting and assembly machinery (SAM) protein import complex to form the larger mitochondrial intermembrane space bridging complex (MIB) at inner-outer membrane contact sites (Ott et al., 2012, 2015; Guarani et al., 2015; Huynen et al., 2016; Kozjak-Pavlovic, 2017). The organization and function of the MICOS and MIB complexes has been thoroughly reviewed elsewhere (Zerbes et al., 2012b; Pfanner et al., 2014; Kozjak-Pavlovic, 2017; Rampelt et al., 2017). We will therefore focus on Mic60 and its potential role in neurodegenerative disease and PD.

Mic60 is a key component of both the MICOS and MIB complexes, interacting either directly or indirectly with the other known components of these complexes (Xie et al., 2007; Harner et al., 2011; von der Malsburg et al., 2011; Ott et al., 2012), and is possibly the oldest evolutionarily conserved component of this structural system (Huynen et al., 2016). Loss of Mic60 leads to destabilization and even loss of MICOS and MIB components (Ott et al., 2015). Further, Mic60 analogs appear to be highly conserved and expressed in all cells containing mitochondria—including plant, yeast, and animal cells—as would be predicted for a protein critical for mitochondrial functions (Odgren et al., 1996; Gieffers et al., 1997; Munoz-Gomez et al., 2015a,b, 2017; Michaud et al., 2016; Wideman and Munoz-Gomez, 2016; Tarasenko et al., 2017).

Multiple studies have now shown that Mic60 is essential for maintaining mitochondrial structure and respiration (John et al., 2005; Rabl et al., 2009; Mun et al., 2010; von der Malsburg et al., 2011; Bohnert et al., 2012; Yang et al., 2012, 2015; Ott et al., 2015; Li et al., 2016; Van Laar et al., 2016; Tsai et al., 2017, 2018). Mic60 loss detrimentally affects cellular viability, especially in response to stress. Viability may be affected by the rearrangement of mitochondrial cristae, impaired mitochondrial respiration, impaired homeostasis, impaired fission and fusion, and disrupted protein import associated with Mic60 deficiency (John et al., 2005; Rabl et al., 2009; von der Malsburg et al., 2011; Bohnert et al., 2012; Zerbes et al., 2012a; Yang et al., 2015; Li et al., 2016; Van Laar et al., 2016). Many of these effects appear to be associated with the reorganization of the mitochondrial membrane structures and protein complexes (Friedman et al., 2015; Eydt et al., 2017; Kozjak-Pavlovic, 2017). In addition to respiratory deficiency and dynamics dysfunction, loss of Mic60 is also linked with mitochondrially associated apoptosis. Yang et al. (2012) demonstrated that reduction of Mic60 expression in HeLa cells resulted in a remodeling of mitochondrial cristae, correlating with increased release of cytochrome c and decreased cell viability in response to apoptosis inducers (Yang et al., 2012). Mic60 knockdown has also been shown to trigger increased calpain activity and apoptosis-inducing factor (AIF) – poly(ADP-ribose) polymerase (PARP) dependent apoptosis in H9c2 myoblasts and HEK 293 cells (Madungwe et al., 2018). Of note, Rossi et al. (2009) found that mitochondrial localization of PARP-1, which they found also regulates mitochondrial DNA (mtDNA) integrity, is dependent on an interaction with Mic60. Multiple studies have now found that suppressed Mic60 affects mtDNA stability, leading to aberrant nucleoid formation, accumulated mtDNA damage, and attenuated mtDNA transcription (Rossi et al., 2009; Yang et al., 2015; Li et al., 2016), and potentially further impairing mitochondrial function. These functions of Mic60 become particularly relevant to PD, where Complex I dysfunction, ROS production, lipid membrane integrity, and hindered mitochondrial quality control are major drivers of pathogenesis. Thus, the stability of Mic60 becomes a key issue in maintaining mitochondrial and cellular health, particularly in cells such as neurons that highly utilize their mitochondria.

## Mic60 Is a Target for Altered Expression and Oxidative Modification During Cellular Stress

Mic60 abundance is highly susceptible to oxidative stress (Magi et al., 2004; Van Laar et al., 2008), which is of particular relevance given that the mitochondrial environment produces high levels of ROS. Exposure to ROS-generating photodynamic therapy, a cancer-treatment method, demonstrated a marked decrease in Mic60 protein levels in cultured HL60 and MCF-7 cells (Magi et al., 2004; Kratassiouk et al., 2006). HL60 cells exposed to the apoptosis-inducing compound homoharringtonine (HTT) showed an initial decrease in Mic60 mRNA expression, followed by a rapid increase (6-fold) in mRNA expression within 6 hrs of treatment, one of only a few genes detected to behave in this manner (Jin et al., 2004). Such a response may suggest that the cells are attempting to recover following a toxic insult. Along this line, Navet et al. (2007) found that expression of Mic60 is significantly increased, along with altered expression of other mitochondrial proteins, in rat brown adipocyte cells during acclimation to colder temperatures, which requires high-energy usage. Of relevance to PD pathogenesis, we demonstrated that Mic60 abundance was significantly decreased in isolated rat brain mitochondria following exposure to DA quinone, as well as in mitochondria isolated from PC12 cells exposed to exogenous DA (Van Laar et al., 2008).

In addition to regulation of Mic60 expression and abundance, the protein itself is also highly susceptible to oxidative modification under stress. Suh et al. (2004) found that exposure of human hepatoma cells to alcohol led to oxidation of Mic60 cysteine residues (Suh et al., 2004). Taylor et al. (2003) examined normal human cardiac tissue mitochondria for oxidative modification of tryptophan residues and found oxidation of selective Mic60 tryptophan residues, suggesting “hot spots” of oxidative susceptibility (Taylor et al., 2003). Mic60 was also found to be carbonylated in kainic acid excitotoxicity-induced neuronal injury in hippocampal cells (Furukawa et al., 2011). Recently, a study found that Mic60 in the brains of aged rats exhibited an age-related increase in oxidative sulfonation of cysteines, with implications for declining neuronal mitochondrial function with age (Yang X. et al., 2018). As discussed in greater detail below, we demonstrated that DA quinone covalently modifies Mic60 in isolated rat brain mitochondria (Van Laar et al., 2009). These studies demonstrate that Mic60 protein and protein levels are highly susceptible to oxidative stress, including PD-relevant oxidative stress. While the consequences of Mic60 protein loss are well documented, the functional consequences of Mic60 oxidative modifications are not known. Modifications that interrupt critical protein-protein interactions could significantly impair Mic60 and MICOS function.

Mic60 has also been found to exhibit other post-translational modifications that potentially regulate its function. In a rat model of traumatic brain injury, Mic60 was found to undergo poly ADP-ribosylation, though the significance of this modification is undetermined (Lai et al., 2008). Studies have now shown that Mic60 function is regulated under cellular and mitochondrial stress via direct phosphorylation by protein kinase A (PKA) and PD-associated mitochondrial kinase PINK1, altering interaction between Mic60 and other proteins and its inner-membrane structural shaping function (Akabane et al., 2016; Tsai et al., 2018). The specific effects of this phospho-regulation and their relevance to PD are further discussed below.

## A Role for Mic60 in Mitochondrial Dynamics and Implications for Neurodegeneration

In addition to respiratory regulation and apoptosis signaling, Mic60 also appears to be a key player in regulating the mitochondrial dynamics of fission, fusion, transport, degradation, and biogenesis (Weihofen et al., 2009; Ding et al., 2015; Li et al., 2016; Van Laar et al., 2016; Akabane et al., 2016; Cho et al., 2017; Tsai et al., 2017, 2018). Balance of these dynamic properties is critical for mitochondrial health and cellular viability, particularly in mitochondria-dependent neurons (Van Laar and Berman, 2013). We were the first to show a functional relationship between mitochondrial fission-fusion dynamics and Mic60 abundance in neurons, demonstrating that increased Mic60 suppressed mitochondrial fission in neurites, leading to longer neuritic mitochondria (Van Laar et al., 2016). Loss of Mic60 in mammalian cell lines was associated with decreased levels of multiple fission and fusion proteins, and corresponding lower fission and fusion rates (Ding et al., 2015; Li et al., 2016). Mic60 loss also impaired mtDNA nucleoid formation and mtDNA transcription (Li et al., 2016), key steps in mitochondrial division and biogenesis. Recent evidence shows that Mic60 also regulates transport. Mic60 associates with a complex containing Miro, a mitochondrial outer membrane protein that regulates kinesin-based mitochondrial anterograde axonal transport (Wang and Schwarz, 2009; Weihofen et al., 2009). Recently, Tsai et al. (2017) demonstrated that Mic60 loss in *Drosophila* was associated with a loss of Miro, leading to an arrest of neuronal mitochondrial movement. This was also associated with functional and structural disruption of neuromuscular junction synapses, suggesting that Mic60 loss has a detrimental impact on axons and axonal mitochondrial health (Tsai et al., 2017).

Mic60 also interacts with proteins involved directly in the general regulation of mitochondrial dynamic processes, as well as ones linked to neurodegenerative diseases. This places it in a unique position to regulate the response to PD-relevant stress. Mic60 interacts with the optic atrophy-linked protein OPA1 (Darshi et al., 2011; Banerjee and Chinthapalli, 2014; Barrera et al., 2016; Glytsou et al., 2016; Hering et al., 2017). OPA1 regulates fusion of the inner mitochondrial membrane between two mitochondria and has been implicated in cristae remodeling (Frezza et al., 2006). Evidence suggests that the relationship between Mic60 / MICOS complex and OPA1 is key in regulating mitochondrial fusion (Cho et al., 2017). However, there are conflicting results as to whether OPA1 plays an integral role in the function of Mic60 and the MICOS complex to organize cristae junctions (Barrera et al., 2016; Glytsou et al., 2016). Mic60 has also been associated with PINK1, a protein involved in regulating mitochondrial homeostasis and linked to a familial form of PD (Weihofen et al., 2009; Akabane et al., 2016; Tsai et al., 2018). This association is discussed in further detail below. The effects of Mic60 specifically on neuronal mitochondrial dynamics, along with the interactions of Mic60 with regulators of mitochondrial dynamics, support that Mic60 may play an important role in maintenance of neuronal health, and potentially in neurodegenerative pathogenesis.

## Evidence Associates Mic60 With PD Pathogenesis

With such an important role in mitochondrial function, Mic60 is likely to be a key player in the health of post-mitotic mitochondria-dependent neurons, especially in times of stress. Indeed, Mic60 has previously been linked to neurological disorders, including fetal Down syndrome (Bernert et al., 2002; Myung et al., 2003), seizure (Omori et al., 2002; Furukawa et al., 2011), schizophrenia (Millar et al., 2005; Park et al., 2010; Atkin et al., 2011), Amyotrophic Lateral Sclerosis (ALS) (Fukada et al., 2004), optic atrophy (Abrams et al., 2015; Abrams et al., 2018), and neurodegeneration in animal models (Wang et al., 2008). While the evidence for Mic60 and these neurological disorders may represent a general effect of aberrant Mic60 expression, protein modification, or protein-protein interactions on neuronal health, little evidence has directly implicated Mic60 itself as a major causative factor in these diseases. However, emerging evidence from multiple studies has begun to demonstrate a strong association between Mic60 and the pathogenic processes in PD.

### Mic60 as a Target of Covalent Modification by DA Quinone, and Loss in DA and MPTP Toxicity

Studies have demonstrated that Mic60 protein abundance is affected by PD-relevant toxicants *in vitro*. We identified Mic60 in a proteomic screen for mitochondrial proteins sensitive to oxidative stress in the DA oxidation model of PD. Following exposure of isolated rat brain mitochondria to DA quinone, Mic60 was found to be covalently modified by DAQ and its abundance was decreased by more than half, amongst the most decreased of all proteins identified in our study (Van Laar et al., 2008, 2009). Similarly, in we found that Mic60 abundance was decreased and the protein covalently modified by DA in neuronally differentiated dopaminergic cells exposed to exogenous DA (Van Laar et al., 2008, 2009). Consistent with our findings, Burte et al. (2011), found that exposing neuronally differentiated dopaminergic mouse N2a cells to MPTP also lead to decreased levels of Mic60 expression (Burte et al., 2011).

While the effects of loss of Mic60 on mitochondrial function have been well demonstrated, the specific effects of covalent DA modifications to Mic60 on cellular health are not clear. Notably, we observed larger molecular weight bands immunopositive for Mic60 in Western analysis of DA-treated cells, suggesting DA oxidation-induced SDS-insoluble interaction of Mic60 proteins. As cysteines are typically utilized in protein-protein interactions, it is likely that such modifications disrupt the ability of Mic60 to properly interact with and regulate the MICOS complex.

### Mic60 Loss Exacerbates Vulnerability to PD Toxicants, and Overexpression Protects Against Models of PD

We recently demonstrated that a modest loss of Mic60 (-30%) did not affect the basal cellular viability of dopaminergic neuronal cells, but significantly exacerbated cellular vulnerability to the PD-relevant toxicant exogenous DA. (Van Laar et al., 2016). This suggests even a slight loss can greatly impact cellular response to mitochondrial stress. Conversely, Mic60 overexpression in dopaminergic cells *in vitro* increased mitochondrial respiratory capacity and promoted cellular survival in response to both toxicants rotenone and exogenous DA (Van Laar et al., 2016). This was the first demonstration that Mic60 loss increased the vulnerability of dopaminergic neuronal cells and the first demonstration that increased Mic60 expression in dopaminergic cells was protective in a toxicant model of PD. In a recent study, Mic60 also appeared to be protective in a genetic model of PD (Tsai et al., 2018).

As noted previously, perhaps the most dramatic characteristic associated with Mic60 loss is the severe reorganization of the mitochondrial cristae structure, resulting in concentric ring-like “onion” structures, or whorls (John et al., 2005; von der Malsburg et al., 2011), and these have recently been seen *in vivo* in Mic60 mutant flies (Tsai et al., 2017, 2018). Similar effects of mitochondrial structural dysregulation have been noted in other models of PD. PINK1 and Parkin knockout flies exhibit mitochondria with abnormal morphology and disorganized internal structures, including whorls (Park et al., 2006; Deng et al., 2008; Poole et al., 2008; Tsai et al., 2018).

Excitingly, a recent study demonstrated that Mic60 overexpression was protective in a genetic PD model, the PINK1 knockout model in flies (Tsai et al., 2018). Mic60 overexpression rescued the mitochondrial cristae disorganization that is exhibited in this PD model, in addition to protecting against multiple other parkinsonian phenotypes, including mitochondrial complex 1 activity deficits, ATP levels, DA neuron degeneration, and behavioral defects. In fact, Mic60 overexpression rescued the PINK1 PD phenotype to a greater extent than overexpression of Parkin, another familial PD-associated protein that functions downstream of PINK1 (Tsai et al., 2018). As Mic60 is a known interactor of PINK1 (Weihofen et al., 2009; Akabane et al., 2016; Tsai et al., 2018), this finding strengthens the relationship between multiple genetic forms of PD and a shared, convergent pathway in regulating mitochondrial function.

In human studies, Tsai et al. identified rare mutations in the mitochondrial targeting sequence of Mic60, some of which were associated with PD patients (Tsai et al., 2018). The mutations were shown to impair the mitochondrial targeting of Mic60 and resulted in disrupted mitochondrial structure when expressed in *Drosophila* (Tsai et al., 2018). These studies suggest a possible genetic link between Mic60 deficiency and PD risk.

### Mic60 Interacts With PINK1 and Is Regulated via Phosphorylation by PINK1 and PKA: Implications for PD Pathogenesis

Previous studies have found Mic60 interacts with PINK1, a protein whose recessive mutations cause familial PD (Weihofen et al., 2009; Akabane et al., 2016; Tsai et al., 2018). New evidence suggests that the Mic60 interaction is regulated via phosphorylation of Mic60, affecting its interaction with PINK1 and MICOS proteins. Both protein kinase A (PKA) and PINK1 itself have now been demonstrated to phosphorylate Mic60 directly and influence function (Akabane et al., 2016; Tsai et al., 2018), which carries implications for Mic60 having a key role in PD pathogenesis.

Protein kinase A is a tetrameric holoenzyme (Corbin et al., 1978), and is sub-cellularly targeted, where binding of cyclic-AMP (cAMP), a major activator of PKA, releases catalytic subunits to act in several downstream pathways (Herberg et al., 2000; Paulucci-Holthauzen et al., 2009; Christian et al., 2011), some of which are directly relevant to PD pathophysiology. Mitochondrially localized PKA (PKAmt) has been shown to phosphorylate several targets involved in mitochondrial homeostasis and function, regulating their function, including subunits of Complex I (Papa et al., 2001; Valsecchi et al., 2013), the pro-apoptotic protein BAD (Martin et al., 2005), and the mitochondrial fission protein DRP1 (Chang and Blackstone, 2007), and promote mitochondrial function under stress and even blunting mitophagy (Dagda et al., 2011). But recently, direct links to PD neurodegeneration were suggested by finding that PKAmt affects the stability of the PINK1-Parkin complex at the mitochondria via its phosphorylation of MICOS proteins Mic60 and Mic19, thus potentially regulating Parkin-mediated mitophagy of damaged mitochondria. Ackbane et al. found that the phosphorylation status of Mic60 regulates the stability of PINK1 upstream of the PINK1-Parkin mitophagy pathway (Akabane et al., 2016). Specifically, PKA-mediated phosphorylation of Mic60 at serine 528 (S^∗^528) negatively affects MICOS complex assembly and inhibits Mic60 interaction with PINK1. This prevents the stabilization of PINK1 on the surface of damaged mitochondria, thereby inhibiting Parkin recruitment (Akabane et al., 2016). They also found that Mic19, another important MICOS component, was similarly regulated by PKA phosphorylation. These results reinforce the role of PKA signaling in regulating mitochondrial function and homeostasis, clearly defining it as an essential component and regulator of cellular metabolism.

Evidence suggests that in addition to PKA, PINK1 itself may directly phosphorylate Mic60 and thus regulate function of the MICOS complex. PINK1 is a nuclear-expressed mitochondrially targeted kinase first identified as an autosomal recessive form of juvenile-onset PD (Valente et al., 2004). Individuals possessing homozygous or compound-heterozygous mutations for PINK1 exhibit mood and cognitive dysfunction similar to sporadic and PDD/LBD (Gandhi et al., 2006; Steinlechner et al., 2007; Feligioni et al., 2016) and are at increased risk for early onset Parkinson’s disease. Under basal conditions, PINK1 is imported into the mitochondria and processed by matrix processing peptidase (MPP) and presenilin-associated rhomboid-like (PARL) (Jin et al., 2010; Deas et al., 2011; Greene et al., 2012), then released post-processing into the cytosol for further downstream signaling (Dagda et al., 2014; Steer et al., 2015) and degradation (Yamano and Youle, 2013). *In vivo* and *in vitro* studies have shown PINK1 is neuroprotective (Haque et al., 2008; Dagda et al., 2014; Khalil et al., 2015; Steer et al., 2015). Until 2007 PINK1 had only been associated with mitochondrial oxidative stress and dysfunction (Exner et al., 2007; Gautier et al., 2008; Weihofen et al., 2008). Then in 2008, Poole et al. performed a series of experiments in *Drosophila* that identified PINK1 and Parkin as major players in mitochondrial fission, fusion, and morphology (Poole et al., 2008). Shortly thereafter in 2009, Weihofen et al. (2009) demonstrated that PINK1 interacts with MIRO and Milton placing PINK1 in a position to regulate mitochondrial trafficking. In this study, Mic60 was also found to associate in a complex with PINK1. This seminal work was the first to demonstrate that Mic60 may play a role in the observed changes in PINK1-deficient cells. However, how PINK1 and Mic60 interact would remain elusive for nearly 10 years.

In 2010, multiple groups established that PINK1 was stabilized on the mitochondrial outer membrane (OMM) following a collapse of the mitochondrial transmembrane potential, leading to accumulation of PINK1 on the OMM (Matsuda et al., 2010; Narendra et al., 2010; Vives-Bauza et al., 2010). Once PINK1 aggregates on the OMM, it interacts with and phosphorylates both Parkin and Ubiquitin to initiate mitophagy (Kondapalli et al., 2012; Kane et al., 2014; Lazarou et al., 2015). PINK1 has since been extensively studied for its role as a sensor of mitochondrial damage and in inducing mitophagy (Nguyen et al., 2016). Although the PINK1-Parkin mitophagy pathway can be activated under stress conditions *in vivo* (Pickrell et al., 2015), more data are emerging that PINK1 is in some cases dispensable for mitophagy and that PINK1 has other distinct and uncharacterized functions (Lee et al., 2018; McLelland et al., 2018; Yang W. et al., 2018).

In this regard, recent work has shown that Mic60 is a substrate of PINK1, providing evidence that PINK1 may directly alter mitochondrial architecture (Tsai et al., 2018). Tsai et al. showed that PINK1 is necessary for mitochondria to maintain cristae junctions in *Drosophila*, and that this function is mediated by PINK1 directly phosphorylating Mic60. Phosphorylation by PINK1 stabilized the oligomerization of Mic60 and increased cristae junctions (Tsai et al., 2018). Further, Mic60 overexpression could rescue multiple phenotypes of PD model PINK1 knockout flies, as mentioned previously, demonstrating that PINK1 modulates the ability of Mic60 to regulate cristae structure and mitochondrial function (Tsai et al., 2018). This regulatory interaction was found to be preserved in human cells, as well, and may be influenced by increased energy demands depending on cell type and/or the location of the mitochondrion within the cell (Tsai et al., 2018).

This phospho-regulation of Mic60 may provide insight into the cellular control of mitochondrial function under various bioenergetic and stress conditions. The evidence suggests that while PKA phosphorylation appears to disrupt the function of Mic60 to interact with and stabilize PINK1 on the OMM, phosphorylation by PINK1 increases Mic60 stability within the MICOS complex, allowing for increased mitochondrial function (Figures 1B–G). This suggests a dual regulation based on the stress status of the cell and on which stress pathways are activated. Interestingly, overexpression of PKA has been observed to rescue PINK1 deficiency, so it is possible that PKA is in part regulating Mic60 in a manner similar and parallel to PINK1 (Dagda et al., 2011; Kostic et al., 2015) The significance of these pathways to PD pathology, or their relevance to one another, remains to be elucidated.

A question regarding these findings is how these systems work in balance to regulate mitochondrial structure and degradation. In the studies by Akabane et al. (2016), PKA phosphorylation of Mic60 affected PINK1-Parkin recruitment, but not mitochondrial cristae structure. This observation occurred despite the decrease in PINK1-Mic60 interaction and the disruption of the MICOS complex. This seems to be in opposition to the observations in *Drosophila* by Tsai et al. (2018), where disrupted PINK1-Mic60 interaction dramatically interrupted Mic60 oligomerization and cristae organization. However, Tsai et al. also found that the function of Mic60 in maintaining mitochondrial structure is, at least in part, independent of PINK1, as overexpressing Mic60 compensated for the loss of PINK1 on cristae organization in *PINK1-null* flies (Tsai et al., 2018). Thus, any possible structural regulation effects of Mic60 phosphorylation status at its PKA-phosphorylation sites may be influenced by the level of overexpression of the Mic60 protein in the studies by Akabane et al. More studies should be conducted to definitively address these discrepancies.

Another issue is whether these pathways are relevant to all tissues, or even all species. Tsai et al. noted differences in cristae structure depending on the cell type or subcellular localization of the mitochondria, suggesting the possibility of differential regulation of cristae structure proteins depending on local energy demands (Tsai et al., 2018). While Mic60 itself is shown to be a highly conserved crucial component for mitochondrial structure across species, its phospho-regulation may not be. While the PINK1 phosphorylation sites on Mic60 seem to be preserved across vertebrate and invertebrate species (Tsai et al., 2018), the PKA site appears absent in *Drosophila* and *C. elegans* (Akabane et al., 2016). On the other hand, the PKA site is conserved across all examined species in another MICOS complex protein, Mic19 (Akabane et al., 2016). This variation of phosphorylation-sites carries implications for the evolution of MICOS complex regulation across species and merits further investigation.

## Mic60 as a Therapeutic Target for PD and Other Diseases

The known functions of Mic60 and the reported findings on altered Mic60 expression allow us to speculate on a potential role for this protein in neuropathogenesis. The mitochondrial cristae structures are known to undergo reorganization in times of increased energy demands, cellular stress, and apoptosis (Mannella et al., 2001, 2013; Scorrano et al., 2002; Mannella, 2006). It is likely that Mic60 is participating in this reorganization due to oxidation- or phosphorylation-induced alterations affecting its functions within the MICOS complex. Evidence now exists for this process to be regulated by direct phosphorylation. However, excess oxidative stress may either directly modify Mic60, which could alter its structure, affect its ability to be phosphorylated, or target it for degradation, further allowing for detrimental cristae destabilization and reorganization. As the MICOS complex also regulates mitochondrial protein import (Xie et al., 2007; von der Malsburg et al., 2011; Bohnert et al., 2012), a loss of Mic60, and thus mitochondrial protein biogenesis, may further hamper efforts of the mitochondrion to recover from excessive protein damage and loss, setting up a deadly cycle of ROS generation and oxidative protein and lipid damage, ultimately leading to mitochondrial collapse. In dopaminergic neurons, this effect could be amplified by DA oxidation and covalent modifications to Mic60, contributing to the selective vulnerability of these neurons in PD.

The crucial role of Mic60 in regulating so many aspects of mitochondrial homeostasis, combined with a susceptibility to oxidative modification and stress-induced loss of abundance, make it an attractive target for investigating therapeutic strategies for PD and other diseases. Our own evidence suggests that increased Mic60 availability in dopaminergic cells is protective against PD-relevant stressors *in vitro*, and Tsai et al. demonstrated that Mic60 overexpression can rescue PD phenotypes *in vivo* in PINK1-mutant *Drosophila* (Van Laar et al., 2016; Tsai et al., 2018). Given the importance of Mic60 and the reliance of neurons on mitochondrial health, a strategy targeting Mic60 may provide protection in multiple neurological disorders, including PD.

Given the central role of Mic60 in mitochondrial homeostasis and function, it is not surprising that Mic60 upregulation may protective in disorders other than neurologic disorders. Studies in patients and models have also linked Mic60 with obesity, diabetes, osteoporosis, and cardiac dysfunctions (Baseler et al., 2011; Guo et al., 2013; Gutierrez-Salmean et al., 2014; Gorr and Wold, 2015; Lindinger et al., 2015; Wang et al., 2017; Lv et al., 2018), and upregulation of Mic60 has shown to be protective in models of diabetes and osteogenesis (Thapa et al., 2015; Lv et al., 2018). Thus, there is likely a widespread effect of improving mitochondrial stability in general via Mic60 upregulation. That being said, the direct links between Mic60 and multiple specific PD gene products suggest the likelihood of a more specific role in PD neurodegeneration. Further study is needed to expound upon the protective findings of Mic60 overexpression and examine the role and function of increased Mic60 in cellular and neuronal health.

## Conclusion

The crucial role played by Mic60 at the intersection of mitochondrial structure, function, and homeostasis make it an exciting target to explore for therapeutic intervention in diseases associated with mitochondrial dysfunction, such as PD. Multiple studies have now linked Mic60 deficiency with PD-relevant cellular stress and have clearly placed Mic60 as a player in the PD-associated PINK1-Parkin cellular pathway. Further research into the role of Mic60 in PD may yield exciting new avenues for disease-altering therapeutic interventions.

## Author Contributions

VV and SB contributed to the initial organization of the review. VV, SB, PO, and TH contributed to the writing of multiple sections and substantive revisions.

## Conflict of Interest Statement

The authors declare that the research was conducted in the absence of any commercial or financial relationships that could be construed as a potential conflict of interest.
